# A Deep Dive Into Instagram's Top Skinfluencers

**DOI:** 10.2196/49653

**Published:** 2023-11-10

**Authors:** Hadley Johnson, Claire Herzog, Rob L Shaver, Sara A Hylwa

**Affiliations:** 1 University of Minnesota Medical School Minneapolis, MN United States; 2 University of South Dakota Sanford School of Medicine Sioux Falls, SD United States; 3 Park Nicollet Contact Dermatitis Clinic HealthPartners Institute Minneapolis, MN United States; 4 Department of Dermatology University of Minnesota Minneapolis, MN United States

**Keywords:** Instagram, skinfluencer, skinfluencers, skin care, social media, general dermatology, training, dermatology, social media, influencer, influencers, skin

## Abstract

We characterized skinfluencers from various training backgrounds and compared their posts on Instagram featuring skin care products.

## Introduction

Skinfluencers are online personalities who share information on skin care routines and products on social media. Launched in 2010, Instagram is a free photo- and video-sharing app [1]. With over 800 million users, Instagram has become a vital business platform for budding skinfluencers—those who share information on cutaneous health [1]. However, medical credentials are not required to share skin care advice online. This poses at minimum a source of confusion and is a risk to patients when inaccurate or low-quality information is shared, which has occurred among hairfluencers [2]. Therefore, the goal of our study was to characterize skinfluencers from various training backgrounds and to compare their Instagram posts featuring skin care products.

## Methods

We identified skinfluencers from 4 different training backgrounds using the following Google search terms: “skinfluencers” and “physicians,” “physician assistants,” “nurse practitioners,” or “aestheticians.” The top 5 skinfluencers with the most Instagram followers from each of the 4 training backgrounds were included in the analysis. We tracked their posts on Instagram for 1 month (March to April 2021) using Microsoft Excel (Microsoft Corp) and collected the following parameters: demographics, follower count, verification status (a badge of authenticity and notability given to select users), and number of posts (total count, posts on skin care products, sponsored and self-promotional posts).

## Results

A total of 20 skinfluencers were identified: 18 (90%) were female and 2 (10%) were male. All skinfluencers were from the United States, with 7 states and 11 cities represented. Physicians had the highest average follower count (n=1.1 million), followed by estheticians (n=523,000). Physician (n=4) and esthetician (n=4) accounts were most frequently verified. Estheticians published the most posts and stories (mean/skinfluencer=490.8), compared to nurse practitioners (mean/skinfluencer=292.6), physicians (mean/skinfluencer=284.6), and physician assistants (mean/skinfluencer=283.2) (Multimedia Appendix 1). Estheticians published the most posts and stories related to skin care products (mean/skinfluencer=70), followed by physicians (mean/skinfluencer=64.4) (Multimedia Appendix 2). Eight skinfluencers published posts on products from their own brands, though physicians had the most self-promotional posts (mean/skinfluencer=10.4). Estheticians had the highest average number of sponsored posts (mean/skinfluencer=1.8), followed by physician assistants (mean/skinfluencer=1.4).

## Discussion

While physicians have a prominent following on Instagram and publish frequently, the greatest volume of skin-related content in this study was shared by those with the least amount of formal medical training. Although estheticians can provide valuable skin care services to patients, their training programs may be limited to only 6 months in duration. This is considerably less than the 8 or more years required for a dermatologist to practice in the United States. Yet, board-certified dermatologists comprise only a small portion (4%) of accounts that share popular dermatologic content on Instagram [3]. This poses a unique opportunity for dermatologists to engage with patients worldwide, as social media has the power to increase access to health information and can lead to behavioral change [4]. Instagram may be a particularly amenable platform for dermatologists, given the visual nature of both the specialty and the platform. However, social media involvement does not come without its pitfalls, especially with regard to self-promotion and sponsorship, which may be unethical [5]. As a result, organizations such as the American Medical Association and the Federation of State Medical Boards have issued guidelines for medical professionals on the proper use of social media [4]. Ultimately, whether dermatologists choose to engage with social media or to remain in the clinical sphere only, it is crucial that they are aware of the influence of social media on patients and of its limitations.

## Appendix

Multimedia Appendix 1 Number of posts versus training background.

Multimedia Appendix 2 Number of posts about skin care versus training background.

## References

[1] Green DD, Martinez R, Kadja A, Evenson L, MacManus L, Dirlbeck S (2018). In a world of social media: a case study analysis of Instagram. Am Res J Bus Manag.

[2] Paiewonsky B, Heinen N, Hordinsky M, Sadick N, Farah RS (2023). Hairfluencer social media trends every dermatologist should know in 2021. J Cosmet Dermatol.

[3] Ranpariya V, Chu B, Fathy R, Lipoff JB (2020). Dermatology without dermatologists? Analyzing Instagram influencers with dermatology-related hashtags. J Am Acad Dermatol.

[4] Ventola CL (2014). Social media and health care professionals: benefits, risks, and best practices. P T.

[5] Smith C, George D (2018). When is advertising a plastic surgeon's individual "brand" unethical?. AMA J Ethics.

