# Supplementary figures and images for: A Deep Dive Into Instagram's Top Skinfluencers
Source: JMIR Dermatol. 2023 Nov 10;6:e49653. doi: 10.2196/49653 (PMC10674138; doi:10.2196/49653)

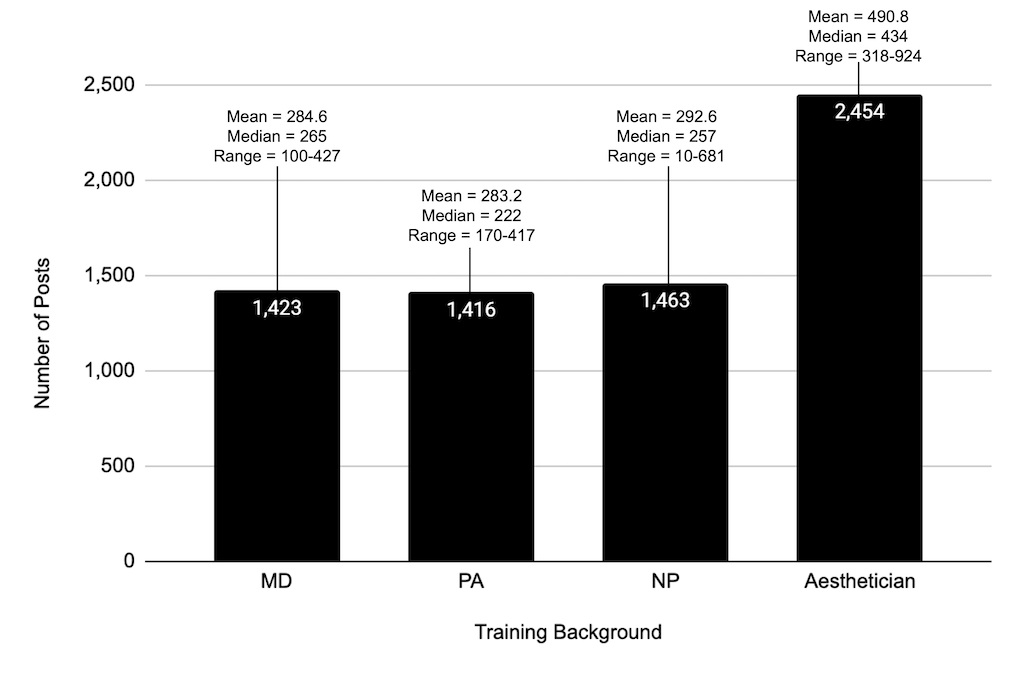

Supplement: Multimedia Appendix 1 [file derma_v6i1e49653_app1.png]

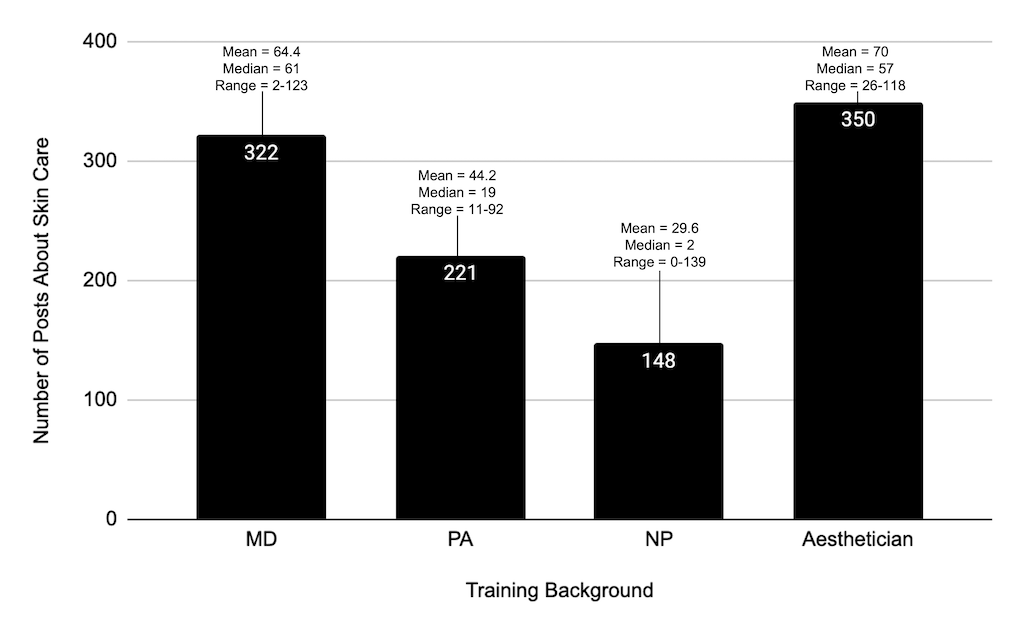

Supplement: Multimedia Appendix 2 [file derma_v6i1e49653_app2.png]
